# Endotracheal tube repositioning and its association with ventilator-associated pneumonia in pediatric intensive care unit: a hospital-based cross-sectional study

**DOI:** 10.1017/ash.2026.10861

**Published:** 2026-09-17

**Authors:** Pakkiresh Reddy, Pradeep Kumar, Lokesh Kumar Tiwari, Arun Prasad, Chandramohan Kumar, Bhabesh Kant Chowdhry, Sweta Sweta

**Affiliations:** 1 Pediatrics, All India Institute of Medical Sciences – Patna, Patna, India; 2 Pediatrics, All India Institute of Medical Sciences – Rishikesh, Rishikesh, India; 3 Neonatology, All India Institute of Medical Sciences – Patna, Patna, India

## Abstract

**Background:**

Ventilator-associated pneumonia (VAP) is one of the most common hospital-acquired infections. The existing literature has revealed several risk factors for the development of VAP, but only a few studies show an association between endotracheal tube (ETT) repositioning and a risk of VAP.

**Objective:**

The Primary objective was to evaluate repositioning of ETT as a risk factor for ventilator-associated pneumonia in children admitted for more than 48 hours for non-pulmonary indications in the pediatric intensive care unit (PICU). The secondary objective was to assess the role of reintubation as a risk factor for VAP in the same cohort.

**Design:**

Hospital-based cross-sectional study.

**Setting:**

Tertiary care facility equipped with pediatric intensive care.

**Participants:**

Children aged less than 15 years.

**Results:**

Repositioning of ETT was done in 11 patients, of whom 8 had VAP. Patients with ETT repositioning had twice the odds of developing VAP, although this was not statistically significant (OR = 2.22; *P* = .2). Reintubation was done in 27 patients, out of which 22 (57.9%) had VAP (*P* < .001), and it increased the risk of VAP by 6.3 times (OR = 6.3; *P* < .001). Multivariate regression analysis revealed that reintubation was the only independent risk factor for the development of VAP.

**Conclusion and relevance:**

Although our analysis did not find ETT repositioning to be an independent risk factor, the observed results indicate that it would be beneficial to look further in larger pediatric cohorts. These results need to be regarded as hypothesis-generating. Prevention of VAP is an important factor in decreasing morbidity and mortality.

**What is already known:** Reintubation is a known risk factor for ventilator-associated pneumonia in the PICU.

**What this study adds:** Repositioning of the endotracheal tube increases the risk of VAP, which requires further research with a larger sample size.

**How this study might affect research, practice, or policy:** Accidental repositioning of endotracheal tubes and unplanned extubation should be minimized to decrease the risk of VAP in ventilated patients admitted to PICU.

## Introduction

Healthcare-associated infections pose a severe risk to patient safety since they are associated with morbidity, extended hospital stays, and death. The use of invasive devices in the Pediatric Intensive Care Unit (PICU), such as central venous lines and mechanical ventilation, leads to an increased risk of Hospital-acquired infections.^1^ Mechanical ventilation forms the core of the treatment of critically ill children in intensive care. One of its complications is the potential for developing pneumonia, often known as ventilator-associated pneumonia (VAP).^2^ VAP is defined as nosocomial pneumonia occurring 48 hours or more after initiation of mechanical ventilation,^3^ and itis the second most common nosocomial infection, the first being bloodstream infection.^1,4,5^ The entry of the microorganism into the sterile lower respiratory tract can occur by following routes: aspiration of oropharyngeal secretions containing pathogens from the sinuses or stomach, spread of microbes from the pleura or hematogenous spread from distant sites, inhalation of contaminated aerosols.^6,7^

Commonly observed risk factors for the development of VAP are aspiration of secretions, use of contaminated equipment, medications, presence of a genetic syndrome, re-intubation, enteral feeding, transport out of the pediatric intensive care unit, and duration of mechanical ventilation.^8^ The presence of an endotracheal tube (ETT) is closely related to the occurrence of VAP. The postulated pathophysiological mechanism suggests that an ETT may inhibit mucociliary clearance, interfere with the cough reflex, and damage the tracheal surface, thus allowing bacteria to enter the lower respiratory tract rapidly.^9,10^ The risk for VAP increases as the length of time, endotracheal tube in situ, increases, especially so in the first two weeks of intubation.^11^ Reintubation is an important factor that increases the risk of VAP, which is shown in many studies. Reintubation results in the aspiration of nosocomial bacteria, which are colonizing the oropharynx. One of the studies showed that patients who underwent reintubation experienced a VAP incidence of 30%, and in non-reintubated patients, it was only 12%.^12^ Till now, no pediatric studies have addressed this question, and differences in susceptibility and care practices limit direct extrapolation from adults.

We hypothesized that endotracheal tube repositioning can increase the risk of VAP in children. Our primary objective was to examine this association in children ventilated for more than 48 hours due to non-respiratory conditions, while the secondary objective was to evaluate the risk of VAP from reintubation. Children with primary respiratory illnesses already carry a higher baseline risk of pneumonia. To avoid this overlap and better examine modifiable risk factors, we limited our study to those intubated for non-respiratory reasons.

## Methods

### Study design

Hospital-based cross-sectional study. Although it was a cross-sectional study, patients were followed up till their final PICU stay.

### Study setting

This was a hospital-based cross-sectional study conducted in the PICU of a multispecialty teaching institute in Eastern India, over one and a half years from September 2021 to February 2023, after the approval of the Institutional Ethics Committee.

### Study participants

Children aged less than 15 years who were intubated for non-respiratory causes for more than 48 hours.

### Sample size estimation

A sample size of 66 was calculated using Statulator.com, a single proportion formula, based on the study on incidence of VAP by Kusahara et al. and considering the total number of intubated patients for non-respiratory causes in our setup.^13^

### Main outcome measures

The primary outcome was the occurrence of VAP, based on Center for Disease Control (CDC) criteria, among the patients who had ETT repositioning.

### Study procedure

Patients aged <15 years, including neonates and children, who required endotracheal intubation for more than 48 hours due to non-respiratory causes and were admitted to the PICU during the study period were consecutively enrolled in the study. Patients with the diagnosis of pneumonia at the time of admission, patients who developed pneumonia within 48 hours of intubation, and to avoid diagnostic overlap, the patients who were intubated for non-respiratory causes were excluded. The point at which the ETT was fixed after intubation, was noted. Details regarding the non-modifiable risk factors for VAP, like any genetic syndromes, neurological impairment, immunodeficiency, airway malformations like tracheoesophageal fistula, and modifiable risk factors like reintubation, repositioning of the tube, duration of mechanical ventilation, bloodstream infections, and use of prolonged antibiotics and steroids were noted.

Participants were carefully observed for any signs of nosocomial infection or VAP, particularly purulent tracheal secretions, fresh or recurring abnormal respiratory sounds on auscultation, increased oxygen requirements or worsening in oxygenation after 48 hours, and body temperature instability. Whether any repositioning or reintubation of ETT was done during mechanical ventilation was also noted.

## Definitions and data collection

- **VAP diagnosis: VAP was diagnosed according to the CDC 2021 criteria (Table 1).**
- **Reintubation:** Reinstitution of ventilatory support within 24–72 hours of extubation, including cases of accidental or unplanned extubation.^14,12^
- **ETT Repositioning:** Defined as any change in endotracheal tube depth compared with the position recorded at baseline. It was categorized as:
  - Intentional repositioning: Physician-mandated correction (e.g., based on chest radiograph).
  - Accidental repositioning: Unplanned displacement due to patient movement, nursing care, or slipping of the tube. Tube position was checked daily at the lip markings and whenever displacement of ETT was suspected; chest radiographs were obtained if clinically indicated.^15^
- **Other Variables:** Demographics, comorbidities, duration of intubation, duration of ventilation, antibiotic use, bloodstream infections, and PICU/hospital stay were documented.

**Figure 1.**
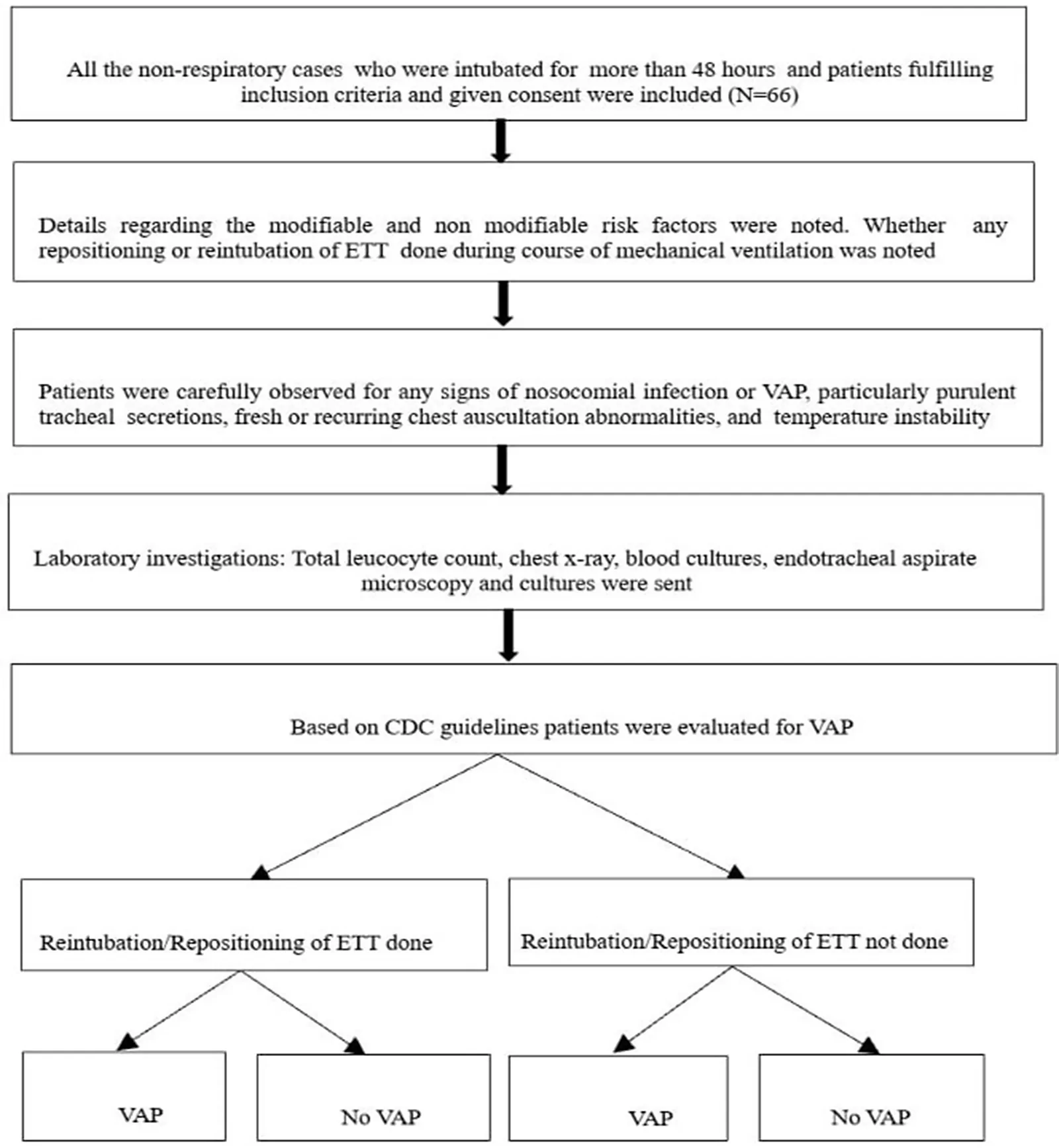
**Study flow.** ETT, Endotracheal tube; CDC, Center for Disease Control; VAP, ventilator-associated pneumonia.

Each patient suspected of VAP underwent a septic screen, which included complete and differential blood counts, blood cultures, tracheal aspirates for cultures, and chest X-rays (Figure 1).

**Table 1.** Centers for Disease Control (CDC) criteria- 2021 guidelines for VAP Table 1 long description.

VAP was diagnosed after 48 hours of mechanical ventilation when the Centers for Disease Control (CDC) criteria- 2021 guidelines were met. According to CDC guidelines, VAP was diagnosed based on these criteria:
1. Clinically defined pneumonia (PNU1)
2. Pneumonia with common bacterial or filamentous fungal pathogens and specific laboratory findings (PNU2)
3. iral, Legionella, and other bacterial pneumonia with definitive laboratory findings (PNU2)
4. Pneumonia in immunocompromised patients(PNU3)

VAP was diagnosed based on the Centers for Disease Control (CDC) guidelines (Table 1). The American Thoracic Society and the Infectious Diseases Society of America recommend non-invasive sampling combined with semiquantitative cultures for the diagnosis of VAP.^16^ This recommendation is supported by data showing no difference in mortality or hospital stay with either invasive or non-invasive sampling methods.^17^ Tracheobronchial aspiration (ie, endotracheal aspirate) was collected by using non-invasive techniques with mucus extractor and semiquantitative cultures were employed to identify VAP. Semiquantitative cultures often indicate whether bacterial growth is heavy, moderate, mild, or absent. We regarded moderate to heavy (>10^5^ CFU/mL) growth as positive. During the hospital stay, the patients were monitored for reintubation or repositioning of ETT. Data from patients with reintubation or repositioning of ETT were compared with those without reintubation or repositioning of ETT to see the risk associated with reintubation or repositioning of ETT in causing VAP. Demographic details, duration of intubation, duration of mechanical ventilation, sedation, stress ulcer prophylaxis, and neuromuscular paralysis to identify other risk factors for the development of VAP were compared. The patients were followed up till recovery, discharge, or death. Outcomes were measured in terms of whether reintubation or repositioning of ETT increases the risk of VAP, length of PICU stay, duration of mechanical ventilation, and patient outcome that is discharge to the ward or home, referral to other facilities, and treatment withdrawl or death.

### Statistical analysis

The data were collected and entered into Microsoft Excel and analyzed using Jamovi-2.3.18.0-win64. The categorical variables were represented as frequency and percentage with a 95% confidence interval. The continuous variables were checked for the normality of the data distribution using a Q-Q plot, and were described as mean ± SD if the data were normally distributed. If the data were not in a normal distribution, it was presented as the median and interquartile range (IQR). Pearson’s χ^2^ test or Fisher’s Exact test was used to infer the association between two categorical variables. Missing data were minimal (<5%) and handled by listwise deletion. The association was found to be significant if the *P* value < .05. Variables with *P* < .1 in univariate analysis (reintubation, repositioning, duration of intubation, hospital stay) were included in a multivariate logistic regression model using a backward stepwise method.

VAP diagnoses were made by trained PICU physicians to limit inter-observer variability. Where diagnostic uncertainty existed, findings were independently reviewed by two senior intensivists to ensure consistency.

## Results

### Patient characteristics

In our study, out of 66 patients, the maximum number of patients, that is 38 (57.6%), were children, 26 (39.4%) were infants, and 2 (3%) were neonates. There were 20 (30.3%) females and 46 (69.7%) males. The majority that is 31 patients had central nervous system infections, 11 had cardiovascular problems, 8 had hematological involvement, 8 had gastrointestinal problems, 5 had sepsis, 1 patient had envenomation, renal involvement, and burns injury each.

### VAP incidence

Among the 66 patients, 38 (57.6%) had VAP which is higher than that reported in previous pediatric studies and 28 (42.4%) had no VAP. Out of 38 patients who had VAP, 13 were diagnosed clinically (PNU1), and 25 had pneumonia with common bacterial or filamentous fungal pathogens and specific laboratory findings (PNU2).

### Endotracheal repositioning and reintubation

Repositioning was done in 11 patients, out of whom eight patients had VAP. Given the limited number of patients in this subgroup (n = 11), the findings should be interpreted with caution. Four patients had repositioning of ETT due to accidental pulling by the patient. Three had it because of displacement of ETT from an original fixed position by the nurse, and four due to correction of malpositioned ETTs as seen on standard chest radiographs by the physician. Reintubation was done in 27 patients, out of which 22 (57.9%) had VAP (*P* < .001) and 5 (17.85%) did not have VAP. Out of 27 patients who were reintubated, 12 (44.44%) had reintubation due to tube blockage, 8 (29.6%) reintubation due to extubation failure, 4 (14.8%) due to accidental extubation, and 3 (11.11%) due to tube leakage (Table 2).

**Table 2.** Baseline characteristics, risk factors association with VAP Table 2 long description.

Variables	VAP (n = 38)	No VAP (n = 28)	*P* value
**Age category**			
Neonate	0	2 (7.1%)	
Infant	17 (44.7%)	9 (32%)	.178
Children	21 (55.3%)	17 (60%)	
**Gender**			
Male	26 (68.42%)	20 (71.42%)	
Female	12 (31.58%)	8 (28.58%)	.930
**Repositioning of ETT**			
Yes	8 (21%)	3 (10.71%)	
No	30 (79%)	25 (89.29%)	.265
**Reintubation of ETT**			
Yes	22 (57.9%)	5 (17.85%)	
No	16 (42.1%)	23 (82.14%)	.001
**Antibiotics before intubation**			
Yes	26 (54.2%)	22 (45.8%)	
No	12 (66.7%)	6 (33.3%)	.36

VAP, ventilator-associated pneumonia; ETT, endotracheal tube; PICU, pediatric intensive care.

## Microbiology profile

Among 66 patients, 25 (37.9%) had positive endotracheal aspirate culture and sensitivity. Out of which, 13 had growth of *Acinetobacter baumanii*, 5 had *Klebsiella pneumoniae*, 4 had Pseudomonas, 2 had *Escherichia coli*, and 1 had Gram-negative coccobacilli and budding yeast. 23 (34.8%) patients had positive blood culture. Out of these 5 had growth of E. coli, 4 had Non-candida albicans, 3 cases of each Pseudomonas and Acinetobacter baumanii, and 2 cases of each Burkholderia, Methicillin-resistant coagulase-negative staphylococci (MRCoNS), yeast-like colonies, and 1 case of each Serratia and Streptococcus. There was no correlation between the organism identified and reintubation or repositioning of ETT.

## Outcome

The outcome of patients were in terms of discharge, LAMA (leave against medical advice), or death. A total of 12 patients were discharged to the ward, out of which six patients had VAP, and the same number had no VAP. Eight were discharged home, among them six had VAP. Fourteen patients were referred to other hospitals or went LAMA, of whom nine had VAP. Among 66 patients, overall mortality was 30 (45.45%), where 15 patients had VAP and no VAP each. The mean duration of PICU stay was 17 (11–32) days in VAP patients and 9 (6–16) days in non-VAP patients (*P* = .004). The mean duration of hospital stay was 21 (12.5–34.5) days in VAP patients and 14.5 (7–21.5) days in non-VAP patients (*P* = .02). The mean duration of intubation in VAP patients was 13 (8–23) days and 7 (4–12) days in non-VAP patients (*P* < .001) (Table 3).

**Table 3.** Outcome of patients and association with VAP Table 3 long description.

Variables	VAP (n = 38)	No VAP (n = 28)	*P* value
**Outcome of the patient**			
Discharge to ward	6 (15.78%)	6 (21.42%)	.33
Discharged to home	8 (21%)	2 (7%)	
Referred to other hospitals/LAMA	9 (23.68%)	5 (17.85%)	
Death	15 (39.47%)	15 (39.47%)	
**Duration of PICU stay, (in days)**	17 (11–32)*	9 (6–16)*	.004
**Duration of hospital stay, (in days)**	21 (12.5–34.5)*	14.5 (7–21.5)*	.02
**Duration of intubation, (in days)**	13 (8–23)*	7 (4–12)*	<.001

*Median IQR.

VAP, ventilator-associated pneumonia; ETT, endotracheal tube; LAMA, left against medical advice; PICU, pediatric intensive care.

### Risk factor analysis

Univariate analysis revealed that patients with VAP had a significantly higher proportion and frequency of reintubation, duration of intubation, and hospital stay. Patients with ETT repositioning had twice the odds of developing VAP, although this was not statistically significant (OR = 2.22; *P* = .2). Reintubated patients had 6.3 times odd of developing VAP (OR = 6.3; 95% CI = 1.97–20.2, *P* < .001). The study participants in whom VAP developed had 1.07 times increased duration of intubation days (OR = 1.07; 95% CI = 1.007–1.14, *P* = .02) and 1.04 times increased duration of hospital stay in VAP patients when compared to non-VAP patients (OR = 1.04; 95%CI = 1.001–1.09, *P* = .04). Multivariate logistic regression analysis revealed that reintubation was the only independent risk factor for the development of VAP (OR = 21.2; 95%CI = 2.23–200, *P* < .001) (Table 4).

**Table 4.** Logistic regression analysis of risk factors associated with VAP Table 4 long description.

Variables	Unadjusted OR (95%CI)	*P* value	Adjusted OR (95% CI)	*P* value
**Reintubation**				
No	1	<.001	1	<.001
Yes	6.3 (1.97–20.2)		21.2 (2.23–200)	
**Repositioning**				
No	1	.2	1	.94
Yes	2.22 (0.5–9.2)		1.08 (0.16–6.7)	
**Duration of Intubation (days)**	1.07 (1.007–1.14)	.02	1.03 (0.9–1.1)	.636
**Duration of hospital stay (days)**	1.04 (1.001–1.09)	.04	1.03 (0.9–1.1)	.423

VAP, ventilator-associated pneumonia; OR, odds ratio; CI, confidence interval.

## Discussion

Our study mainly focuses on determining the risk of the development of VAP due to the repositioning and reintubation of the endotracheal tube. The incidence of VAP in our study was 57.6% (95% 45.8–68.7). In 2015, the CDC conducted a point-prevalence survey, in which 32% of the patients had VAP, and in an Indian study, done by Vijay et al., the incidence of VAP was 38.4%.^18,19^ A higher incidence of VAP could be because of use of highly sensitive CDC clinical criteria where microbiological evidence was not present. Due to the inclusion of critically ill patients requiring prolonged ventilation.

Although ETT repositioning had twice the odds of developing VAP, this was not statistically significant (OR = 2.22; *P* = .2) and was not retained on multivariate analysis. Because of the small sample size in the repositioned group, these findings should be interpreted with due caution and considered exploratory. One of the case-control studies done by Murphy et al. showed that ETT repositioning is an independent risk factor on multivariate logistic regression analysis (OR:3.11; 95% CI = 1.03–9.42, *P* = .04).^15^ Ismail et al. showed that the patients who had ETT repositioning were twice as likely to develop VAP compared to patients who had no history of ETT repositioning (unadjusted OR-2.25, 95% CI = .85–5.97, *P* = .13).^20^ The results obtained in our study were almost identical to the results obtained by Ismail et al. Larger, powered studies are required for further evaluation of repositioning as an independent risk factor for VAP. In a study done by da Silva et al., among patients who had unexplained extubation, 65% had reintubation.^19^ A study done by Bilan and Habibi showed that 14.6% of patients underwent reintubation.^12^ One of the studies showed that the incidence of VAP in reintubated patients and patients without reintubation was 30% and 12%, respectively.^14^ In our study, VAP occurred in 57.9% of reintubated patients compared to 42.1% of non-reintubated patients.

The duration of intubation, PICU stay, and hospital stay is longer in VAP patients, which has been reported in previous studies .^21,22^ In comparison with other studies, our study also had similar results, that is there was an increased duration of mechanical ventilation, PICU stay, and hospital stay in VAP patients compared to non-VAP patients.

A higher mortality rate was seen in our study (45%) is notable. Many of the deaths occurred in patients with multiple comorbidities or prolonged ventilation, suggesting that VAP may have been a marker of illness severity rather than a direct cause. Nevertheless, the high mortality underscores the importance of rigorous infection prevention strategies in the PICU. The strength of this study is that only two studies have been done on the repositioning of ETT as a risk factor for VAP in adults and we did not get any studies on the pediatric population.^15,20^ Our study has several limitations. The cross-sectional study design restricts the ability to establish temporality and causation between ETT manipulation and the onset of VAP. The major limitation of the study was the clinical approach to diagnosis, which is oversensitive, resulting in the diagnosis of VAP when another process would have been responsible for the clinical deterioration Interobserver variability in clinical interpretation could not be fully eliminated despite consensus review by senior intensivists. The relatively small number of patients in the repositioning subgroup (n = 11) limited statistical power to detect significant differences. Finally, as a single-center study, the findings may not be generalizable to all pediatric critical care settings. Here, respiratory cases were not included, which could have given information in that arena, too.

## Conclusion

Reintubation was identified as a significant independent risk factor for VAP in ventilated patients. Although ETT repositioning did not show any significant association, it requires multicenter studies, and findings should be viewed as hypothesis-generating. Further research would be needed to determine the repositioning of ETT as a risk factor with a larger sample size to further establish it. Implementation of standardized airway management and extubation protocols may help to reduce reintubation rates and, in turn, the incidence of VAP in pediatric intensive care units.

## Patient and public involvement

The patient and/or the public were not involved in the design, recruitment, conduct, reporting, or dissemination of plans of the study.

## Table 1. Long description

The textbox outlines the criteria for diagnosing ventilator-associated pneumonia (VAP) based on the Centers for Disease Control (CDC) 2021 guidelines. It specifies four criteria for VAP diagnosis: clinically defined pneumonia, pneumonia with common bacterial or filamentous fungal pathogens and specific laboratory findings, viral, Legionella, and other bacterial pneumonia with definitive laboratory findings, and pneumonia in immunocompromised patients. The text also defines reintubation, endotracheal tube (ETT) repositioning, and other variables such as demographics, comorbidities, duration of intubation, duration of ventilation, antibiotic use, bloodstream infections, and PICU/hospital stay.

Navigate back to Table 1..

## Table 2. Long description

The table compares variables associated with ventilator-associated pneumonia (VAP) and no VAP in patients. It has 4 rows and 4 columns. The columns are labeled Variables, VAP (n = 38), No VAP (n = 28), and P value. The rows are labeled Age category, Gender, Repositioning of ETT, Reintubation of ETT, and Antibiotics before intubation. Under Age category, the data is divided into Neonate, Infant, and Children. Under Gender, the data is divided into Male and Female. Under Repositioning of ETT, the data is divided into Yes and No. Under Reintubation of ETT, the data is divided into Yes and No. Under Antibiotics before intubation, the data is divided into Yes and No. Each category lists the number of patients with VAP and without VAP, along with the corresponding P value.

Navigate back to Table 2..

## Table 3. Long description

A table with 10 rows and 4 columns comparing patient outcomes and association with VAP. The columns are labeled Variables, VAP (n = 38), No VAP (n = 28), and P value. The rows include Outcome of the patient with subcategories Discharge to ward, Discharged to home, Referred to other hospitals/LAMA, and Death. Duration of PICU stay, Duration of hospital stay, and Duration of intubation are also compared. For example, 6 patients with VAP were discharged to the ward compared to 6 without VAP. The duration of PICU stay was 17 days for VAP patients and 9 days for non-VAP patients.

Navigate back to Table 3..

## Table 4. Long description

A table comparing risk factors associated with ventilator-associated pneumonia using univariate and multivariate analysis. The table has four rows and five columns. The columns are labeled Variables, Unadjusted OR (95% CI), P value, Adjusted OR (95% CI), and P value. The rows are labeled Reintubation, Repositioning, Duration of Intubation (days), and Duration of hospital stay (days). Row 1: Reintubation, Unadjusted OR (95% CI) No 1, Yes 6.3 (1.97-20.2), P value .001, Adjusted OR (95% CI) No 1, Yes 21.2 (2.23-200), P value .001. Row 2: Repositioning, Unadjusted OR (95% CI) No 1, Yes 2.22 (0.5-9.2), P value .2, Adjusted OR (95% CI) No 1, Yes 1.08 (0.16-6.7), P value .94. Row 3: Duration of Intubation (days), Unadjusted OR (95% CI) 1.07 (1.007-1.14), P value .02, Adjusted OR (95% CI) 1.03 (0.9-1.1), P value .636. Row 4: Duration of hospital stay (days), Unadjusted OR (95% CI) 1.04 (1.001-1.09), P value .04, Adjusted OR (95% CI) 1.03 (0.9-1.1), P value .423.

Navigate back to Table 4..
